# A comparison of digestive strategies for fishes with different feeding habits: Digestive enzyme activities, intestinal morphology, and gut microbiota

**DOI:** 10.1002/ece3.10499

**Published:** 2023-09-12

**Authors:** Fang Jiao, Lei Zhang, Samwel Mchele Limbu, Hong Yin, Yuqing Xie, Zihan Yang, Zongmin Shang, Lingfu Kong, Hua Rong

**Affiliations:** ^1^ College of Marine Sciences South China Agricultural University Guangzhou China; ^2^ Xiangyang Polytechnic Xiangyang China; ^3^ Department of Aquaculture Technology, School of Aquatic Sciences and Fisheries Technology University of Dar es Salaam Dar es Salaam Tanzania; ^4^ Kunming Customs Technology Center Kunming China; ^5^ College of Animal Science and Technology Yunnan Agricultural University Kunming China

**Keywords:** 16S rDNA, fish digestive enzymes, fish feeding habit, gut intestinal microbiota

## Abstract

Fish feeding habit determines the digestive tract structure and intestinal microflora. However, the relationship between feeding habit, digestive intestinal morphology, and microbial diversity of omnivorous, herbivorous, plankton feeder, and carnivorous fish from the same environment has not been compared. This study compared the digestive enzyme activities, intestinal morphology, and intestinal microflora of omnivorous (*Carassius auratus*), herbivorous (*Ctenopharyngodon idellus*), carnivorous (*Siniperca chuatsi*), and plankton feeder (*Schizothorax grahami*) fishes and predicted the potential functions of specific microflora on different nutrients. Twelve intestine samples were collected from each of the four fishes from Dianchi Lake. The composition and diversity of microbial communities were determined by using high‐throughput sequencing of 16S rDNA. The results showed that the carnivorous fish (*S. chuatsi*) had higher trypsin and pancrelipase activities in the hepatopancreas and enteropeptidase in the intestine, but lower amylase activities in the intestine. The carnivorous fish intestine had more microvilli branches and complex structures than other fish species in the order carnivorous > herbivorous > plankton feeder > omnivorous. The intestinal microflora diversity was higher in the omnivorous fish and followed the order omnivorous > herbivorous > plankton feeder > carnivorous. *Acinetobacter* species and *Bacteroides* species were the most dominant flora in the carnivorous and herbivorous fishes, respectively. *Acinetobacter* species and *Pseudomonas* species might help the host to digest protein, while *Bacteroidetes* species may help the host to digest cellulose. Taken together, feeding habit determines the digestive enzyme activities, intestinal tissue morphology, and differential colonization of fish intestinal flora. The knowledge obtained is useful in feed formulation and feeding practices for the studied fish species.

## Graphical abstract

Feeding habit determines the digestive tract structure and intestinal microflora. However, the relationship between feeding habit, digestive physiology intestinal, and microbial diversity of omnivorous, herbivorous, filter‐feeder, and carnivorous fish reared in the same pond has not been compared. This study compared the digestive enzyme activities, intestinal morphology, and intestinal microflora of omnivorous (*Carassius auratus*), herbivorous (*Ctenopharyngodon idellus*), carnivorous (*Siniperca chuatsi*), and filter‐feeder (*Shizothorax grahami*) and predicted the potential functions of specific microflora on different nutrients.

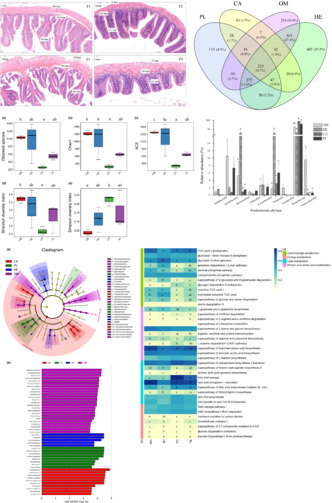

## 1 INTRODUCTION

Fish feeding habits are reflected by their digestive organ, mainly in the intestine. Scholars generally classify fish feeding habits as herbivorous, carnivorous, omnivorous, and filter‐feeders according to the feeding method and food content. The intestine tract is the main site for digestion and nutritional uptake, which has been regarded as a key organ in fish nutrition (Kumar et al., 2005; Wang et al., 2018; Zhou et al., 2021). The fish digestive enzyme activities are closely related to the diet consumed and the fish ability to digest and absorb different nutrients (Bakke et al., 2010; Liu et al., 2021). Evidently, previous studies found herbivorous fish such as Roho labeo (*Labeo rohita*) and Japanese eel (*Anguilla japonica*) had stronger amylase activity compared with carnivorous fish such as Great white catfish (*Wallago attu*) (Agrawal et al., 1975) and rainbow trout (*Oncorhynchus mykiss*) (Hidalgo et al., 1999). Therefore, the influence of feeding habits on digestive enzyme activities is beyond doubt.

The fish feeding habits also affect digestive tract structure and intestinal microorganisms (Li et al., 2019; Meng et al., 2014; Valdes et al., 2018). Interference with intestinal morphology such as muscularis thickness (MT) and villi width (VW) affects nutrient absorption and intestinal microbiota (Limbu et al., 2018). Fish gut microbiota contribute to digestion and affect gastrointestinal tract development and overall fish growth (Clements et al., 2014; Ghanbari et al., 2015). Moreover, feeding habits (Larsen et al., 2014; Meng & Nie, 2019; Roeselers et al., 2011), which determine the consumed diet (Benson et al., 2010; Spor et al., 2011; Sullam et al., 2012), have been reported to shape microbial communities in fish (Larsen et al., 2014; Meng & Nie, 2019; Roeselers et al., 2011). Accordingly, diet has been reported as a dominant source of variation in the microbiota composition of rainbow trout (Desai et al., 2012; Ingerslev et al., 2014). The disruption in intestinal microbiota induced by feeding habit via diet usually affects digestive host functions through disturbance in bacterial digestive enzyme production (Ghanbari et al., 2015). Moreover, certain gut microbiota such as the cellulolytic enzyme‐producing bacterial community, which were isolated from a herbivorous fish intestinal tract, are known to metabolize a remarkable variety of substrates (Li et al., 2016), thereby improving host growth performance. Therefore, several studies have explored gut microbiota manipulation through diet to improve fish growth performance (Fan et al., 2021; Li et al., 2019; Pan et al., 2021). However, studies exploring the relationship among feeding habits, digestive enzyme activities, intestinal structure and gut microbial composition, abundance, and diversity in fish are currently limited. Such a knowledge gap limits our understanding of proper feed formulation and feeding practices in aquaculture.

China is currently the largest producer and consumer of cultured fish (FAO, 2022). Aquaculture production in China includes rearing of Grass carp (*Ctenopharyngodon idellus*), a herbivorous fish (Liu et al., 2017) feeding primarily on aquatic plants, both higher aquatic plants and submerged terrestrial vegetation, but may also eat detritus, insects, and other invertebrates (Cudmore & Mandrak, 2004). The mandarin fish (*Siniperca chuatsi*) is a carnivorous fish also cultivated in China, which mainly feeds on live prey (Shen et al., 2021). Aquaculture production in China also includes species such as Dianchi high‐back crucian carp (*Carassius auratus*), an omnivorous fish (Shi et al., 2017), which consumes organic detritus, filamentous algae, zooplankton, small benthic animals, and pieces of aquatic weeds (Olsén & Lundh, 2016). Moreover, Chinese aquaculture also includes Kunming Schizothoracin (*Schizothorax grahami*), a plankton feeder fish, which is endemic to Yunnan, China (Zhou & Zhang, 2013). Adult *S. grahami* scrapes on water bottom, tree branches, and stones in order to obtain algae species such as blue algae, diatoms, and green algae by using its developed keratin (Zhou & Zhang, 2013). *S. grahami* is the main economic fish produced in Yunnan because of its nutritional value (Zheng et al., 2016). Accordingly, knowledge on the influence of feeding habits on digestive enzymes, intestinal morphology, and microbiota composition is needed for effective feed formulation for these species in order to ensure optimum feed utilization, digestion, and absorption for their sustainable production.

The present study compared the feeding habits, digestive enzymes, intestinal morphology, and intestinal microbiota of *C. idella*, *S. chuatsi*, *C. auratus*, and *S. grahami* as representative fish species for herbivorous, carnivorous, omnivorous, and plankton feeder, respectively. We also predicted the potential functions of specific microflora on different nutrient digestion. The results obtained provide a scientific basis for development of appropriate fish feed formulation.

## 2 MATERIALS AND METHODS

### 2.1 Fish sampling

Ten individual fish for each species (*C. idellus*, *S. chuatsi*, *C. auratus*, and *S. grahami*) were caught by using trolling boats in the Dianchi Lake, Kunming, Yunnan, China. Dianchi Lake is located at latitude 24°23′ N–26°22′ N and longitude 102°10′ E–103°40′ E. The sampling was conducted at latitude 26°03′ N–26°22′ N and longitude 103°100′ E–103°20′ E. During sampling, the Dianchi Lake had a chemical oxygen demand of 29.8 mg/L, total phosphorus concentration of 0.062 mg/L, total nitrogen level 10.6 mg/L, and a water transparency of 0.64 m. The sampled fishes were transported live in plastic bags provided with dissolved oxygen by car to the Aquaculture Laboratory of Yunnan Agricultural University, where they were euthanized by immersing them into 40 mg/L eugenol (Shanghai Reagent). The average weights of the sampled fishes were determined by using a precision weighing scale (Mettler Toledo, XPR10002S, Switzerland) as 1323.60 ± 40.20 g for *C. idellus*, 471.10 ± 23.94 for *S. grahami*, 841.30 ± 34.54 g for *S. chuatsi*, and 350.4 ± 25.98 g for *C. auratus*. These weight data indicate that the fish sampled were all adults.

### 2.2 Determination of digestive enzyme activities

Three fish for each species were dissected carefully and intestine and hepatopancreas were sampled and transferred into an Eppendorf tube. The Eppendorf tube containing the sample was immediately placed into liquid nitrogen. The tubes containing the samples were stored at −80°C until needed for enzyme activities analysis. On analysis days, the hepatopancreas and intestine samples were weighed and mixed with nine times phosphate buffer saline (PBS) (w:v = 1:9), then homogenized by using an electric homogenizer (Ningbo Scientz Biotechnology) in ice bath for 15 s. The resulting homogenate was carefully pipetted and centrifuged at 13,400 g at 4°C for 20 min. Finally, the liquid supernatant was collected for digestive enzyme analysis. The digestive enzyme activities including pepsin (model number A080‐1‐1), trypsin (model number A080‐2‐1), lipase (model number A054‐2‐1), amylase (model number C016‐1‐1), and total protein concentration (model number A045‐2‐2) in the hepatopancreas and intestine were determined by using specific commercial kits (Nanjing Jiancheng Bioengineering Institute) based on instructions from the manufacturer.

### 2.3 Intestinal morphology analysis

Three fish tissues from midgut for each species were collected and prepared for intestinal morphology analysis as described previously (Limbu et al., 2018). The tissues were fixed with 4% paraformaldehyde for 24 h and then dehydrated with 75% absolute ethanol. The tissues were then transferred into xylene (twice) for transparent, immersed into paraffin wax (three times), and embedded and cooled. The intestine tissues were sliced transversely into pieces with approximately 5 to 6 μm, dried, and stained by using hematoxylin and eosin (H & E). The slides were finally examined by using electronic biological microscope KOPPACE at 40× to 1600× (Kopace Technology Co., Ltd.). Villi height (VH), VW, and MT were measured from at least 30 segments for each fish species by using Case Viewer software. Villi height index (VHI), villi width index (VWI), and muscularis thickness index (MTI) were calculated as VH, VW, and MT divided by individual fish body weight^1/3^.

### 2.4 DNA extraction and high‐throughput sequencing analysis of intestinal microbiota

We sterilized the scalpels, tweezers, and scissors by heating them at 180°C for 2 h before they were used for DNA extraction. We also wiped the fish surface, the laboratory bench, and instruments used by using 75% alcohol to disinfect them. Afterward, we collected the gut contents from the remaining four fish samples for each species (three intestines for each fish) and placed them into sterile tubes under sterile conditions. The tubes containing the samples were immediately placed into liquid nitrogen and then stored at −80°C until DNA extraction. The four intestine samples for herbivorous fish (*C. idellus*) were abbreviated as HE, plankton feeder (*S. grahami*) as PL, omnivorous (*C. auratus*) as OM, and carnivorous (*S. chuatsi*) as CA for convenient reporting. These samples were subjected to DNA extraction using the PowerFood Microbial DNA Isolation Kit (QIAGEN Srl) following the manufacturer's instructions. The quality and quantity of the DNA were checked by using gel electrophoresis and a Qubit 4 Fluorometer (Thermo Fisher Scientific). Primers 341F: ACTCCTACGGGAGGCAGCAG and 806R: GGACTACHVGGGTATCTAAT were used to generate the polymerase chain reaction (PCR) amplicons for the 16S rRNA gene V3–V4 region on Illumina sequencing platform (HiSeq™ 2500, Beijing igeneCode Biotech Co., Ltd.).

### 2.5 Bioinformatics analysis

The raw pair‐end readings obtained were subjected to quality‐control procedures by using the quantitative insights into microbial ecology (QIIME, version 1.17). To obtain high‐quality clean reads, raw reads were demultiplexed and filtered for quality based on the methods developed by Fadrosh et al. (2014). Cleaned tags were obtained by FASTP (Chen et al., 2018). The qualified reads were clustered to generate operational taxonomic units (OTUs) at the 97% similarity level by using UPARSE (v7.0.1090) (Edgar, 2013). Chimeric sequences were identified and removed by using UCHIME. The representative phylogenetic affiliation of each 16S rRNA gene sequence from each OTU was then taxonomically classified by using the Ribosomal Database Project (RDP) Classifier (v2.2) against the silva 16S rRNA database using a confidence threshold of 80%. Taxonomic richness and diversity estimators were determined by using a Mothur software.

### 2.6 Prediction of microbiome functions by using bioinformatics analysis

We predicted the gut microbiome functions by using Phylogenetic Investigation of Communities by Reconstruction of Unobserved States (PICRUSt) to elucidate the physiological features and metabolism capability during dietary digestion. To compare the functional categories of microbiota among the four fish species studied (HE, PL, OM, and CA) by PICRUSt analyses, functional profile heatmaps based on 423 categories (Kyoto Encyclopedia of Genes and Genomes, KEGG level‐3) were constructed.

### 2.7 Statistical analyses

All the data for enzyme activities and intestinal morphology were tested for normality and homogeneity of variances by using Shapiro–Wilk and Levene's tests, respectively. Afterward, one‐way analysis of variance (ANOVA) was used to test for statistical differences in the data for enzyme activities and intestinal morphology among the four fish species, representing the four feeding habits. Tukey multiple comparisons test was used to compare for significant differences among the four feeding habits when ANOVA indicated statistical differences. The analysis of enzyme activities and intestinal morphology data was conducted by using SPSS 20.0 (SPSS, Inc.). Results with *p* ≤ .05 were considered significant different. The results obtained are expressed as mean ± standard error of the mean (SEM).

The differences in bacterial phylotype distribution were assessed by using principal component analysis (PCA). The alpha‐diversity indices (the abundance coverage‐based Estimator—ACE, Chao1, Shannon, and Simpson indices) were generated by using Mothur v1.31.2 (http://www.mothur.org/wiki/Calculators). Abundance of microbiota was analyzed by using ACE and Chao1 indices, while microbiota species diversity was assessed by using Shannon and Simpson indices. Linear discriminant analysis effect size (LEfSe) was analyzed by using the R statistical package (v3.1.1).

## 3 RESULTS

### 3.1 Comparative analysis of digestive enzyme activities

The feeding habits affected significantly the digestive enzyme activities of the four fish species studied in the hepatopancreas and intestine (*p* < .05; Table 1). The carnivorous fish (*S. chuatsi*) had higher trypsin and pancrelipase activities in the hepatopancreas and enteropeptidase in the intestine than herbivorous (*C. idella*), omnivorous (*C. auratus*), and the plankton feeder fish (*S. grahami*) (*p* < .05). Moreover, the plankton feeder fish had higher trypsin and pancrelipase activities in the hepatopancreas, but lower entero‐amylase in the intestine than the herbivorous and omnivorous fishes (*p* < .05). The omnivorous fish had significantly higher enteropeptidase activity in the intestine than the herbivorous and plankton feeder fishes (*p* < .05). However, the herbivorous and omnivorous fish species had no significant differences in trypsin and pancrelipase activities in the hapatopancreas (*p* > .05). Similarly, the herbivorous and plankton feeder fishes had no significant difference in enteropeptidase activity in the intestine (*p* > .05).

**TABLE 1:** Digestive enzyme activities of the herbivorous, plankton feeder, and carnivorous omnivorous fish species during the study.

Tissue	Digestive enzyme	Feeding habit			
		Herbivorous	Plankton feeder	Carnivorous	Omnivorous
Hepatopancreas	Trypsin	11.64 ± 0.91^a^	48.44 ± 1.70^b^	114.35 ± 9.46^c^	21.38 ± 1.28^a^
	Pancrelipase	438.99 ± 20.15^a^	722.54 ± 19.14^b^	937.47 ± 10.90^c^	413.93 ± 15.15^a^
	Amylopsin	370.75 ± 20.67^a^	340.46 ± 12.50^a^	166.09 ± 10.69^b^	222.33 ± 16.02^c^
Intestine	Enteropeptidase	28.44 ± 1.70^a^	30.83 ± 1.55^a^	95.03 ± 3.09^b^	50.12 ± 4.26^c^
	Intestinal lipase	1025.02 ± 99.54^a^	372.94 ± 21.91^b^	727.99 ± 34.36^c^	1141.93 ± 100.83^a^
	Entero‐amylase	880.62 ± 31.87^a^	718.25 ± 21.85^b^	420.77 ± 12.89^c^	947.42 ± 48.65^d^

*Note*: Values are mean ± SEM (*n* = 3). Values in the same row with different lowercase letters indicate significant differences (*p* < .05).

Interestingly, the carnivorous fish (*S. chuatsi*) had significantly lower amylopsin activity in the hapatopancreas and entero‐amylase in the intestine than the herbivorous, omnivorous, and plankton feeder fishes (*p* < .05). Similarly, the omnivorous fish had significantly lower amylopsin activity than the herbivorous and plankton feeder fishes in the hepatopancreas (*p* < .05). The herbivorous and plankton feeder fishes had no significant difference in amylopsin activity in the hepatopancreas (*p* > .05). Equally, the herbivorous and plankton feeder fishes had no significant difference in enteropeptidase in the intestine (*p* > .05). The herbivorous and omnivorous had significantly higher intestinal lipase activity than the plankton feeder and carnivorous fish species (*p* < .05). Similarly, the carnivorous fish had significantly higher intestinal lipase than the plankton feeder fish (*p* < .05). The plankton feeder fish had significantly lower entero‐amylase activity than the herbivorous and omnivorous fishes in the intestine (*p* < .05). However, the herbivorous and omnivorous fish species had no significant differences in intestinal lipase and entero‐amylase activities (*p* > .05).

### 3.2 Intestinal tissue morphology

**FIGURE 1:**
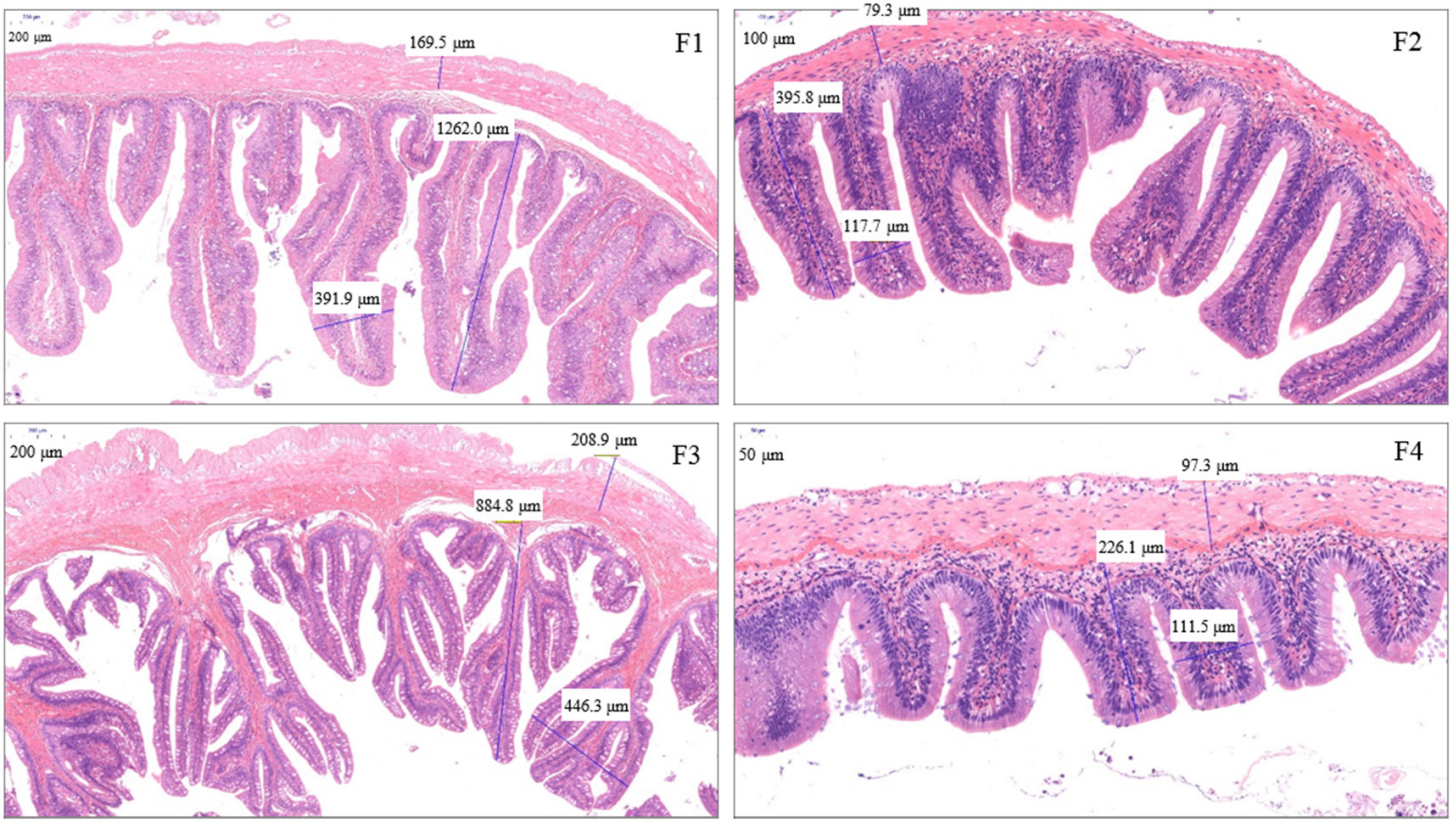
Representative intestinal tissue morphology of the midgut from the herbivorous (F1), plankton feeder (F2), carnivorous (F3), and omnivorous (F4) fish species obtained during the study.

The intestinal microvilli of the carnivorous fish had many branches and complex structures. The order of microvilli complexity was carnivorous > herbivorous > plankton feeder > omnivorous. The VH, VW, and MT differed significantly among the four the fish species (Figure 1; *p* < .05). Herbivorous fish had significantly higher VH and VHI than the plankton feeder, carnivorous, and omnivorous fish species (*p* < .05). Similarly, the carnivorous fish had significantly higher VH and VHI than the plankton feeder and omnivorous fish species (*p* < .05). Likewise, plankton feeder fish had significantly higher VH and VHI than the omnivorous fish species (*p* < .05). On the contrary, the carnivorous fish had significantly higher VW, MT, VWI, and MTI than the herbivorous, plankton feeder, and omnivorous fish species (*p* < .05). Similarly, the herbivorous fish had significantly higher VW, MT, VWI, and MTI than the plankton feeder and omnivorous fish species (*p* < .05). Likewise, the omnivorous fish had significantly higher MT, VWI, and MTI than the plankton feeder fish species (*p* < .05). However, the plankton feeder and omnivorous fish species had no significant difference in VW (*p* > .05) (Table 2).

**TABLE 2:** Intestinal morphology of herbivorous, plankton feeder, carnivorous, and omnivorous fish species obtained during the study.

Parameter measured	Feeding habit			
	Herbivorous	Plankton feeder	Carnivorous	Omnivorous
Villi height (VH, μm)	1284.36 ± 55.24^a^	404.80 ± 10.55^b^	852.06 ± 36.15^c^	223.18 ± 10.15^d^
Villi width (VW, μm)	406.26 ± 12.24^a^	117.00 ± 5.12^b^	443.52 ± 13.06^c^	117.52 ± 7.613^b^
Muscular thickness (MT, μm)	168.28 ± 7.17^a^	80.08 ± 4.50^b^	219.56 ± 9.61^c^	96.38 ± 4.01^d^
Villi height index (VHI)	116.96 ± 5.52^a^	52.38 ± 1.06^b^	90.17 ± 3.67^c^	31.74 ± 1.33^d^
Villi width index (VWI)	36.99 ± 0.93^a^	14.17 ± 0.42^b^	46.94 ± 1.56^c^	17.21 ± 0.74^d^
Muscular thickness index (MTI)	15.32 ± 0.58^a^	10.36 ± 0.62^b^	23.24 ± 1.13^c^	13.71 ± 0.51^d^

*Note*: VHI = VH/BW^1/3^, VWI = VW/BW^1/3^, MTI = MT/BW^1/3^, body weight (BW). Values are mean ± SEM (*n* = 3). Values in the same row with different lowercase letters indicate significant differences (*p* < .05).

### 3.3 Microbial complexity in the gut of the four fish species studied

**FIGURE 2:**
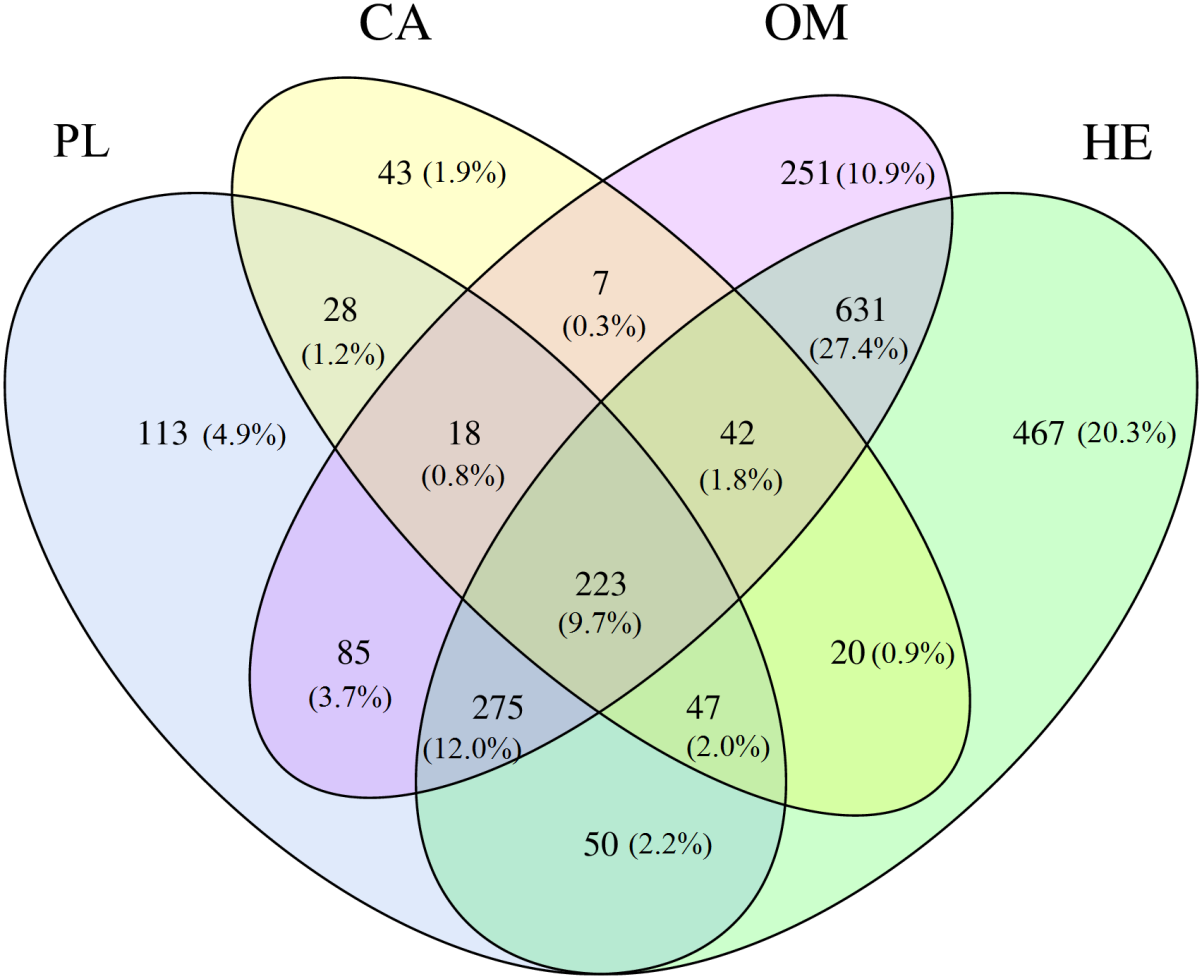
OTUs composition of the four fishes studied with different feeding habits.

A total of 2300 OTUs were obtained for all the four fishes. A total of 223 OTUs were shared by all the four fish species studied (9.7%), while 332 (223 + 42 + 47 + 20) OTUs (14.4%) were shared by the herbivorous and carnivorous fish species (Figure 2). The herbivorous fish had relatively higher number of unique OTUs (467), equivalent to 20.3%, followed by the omnivorous fish (251 OTUs) representing 10.9%, while the plankton feeder fish had 113 OTUs equivalent to 4.9% and the carnivorous fish had only 43 OTUs making up 1.9%.

### 3.4 Microbiota abundance and diversity in the gut of the four fish species studied

**FIGURE 3:**
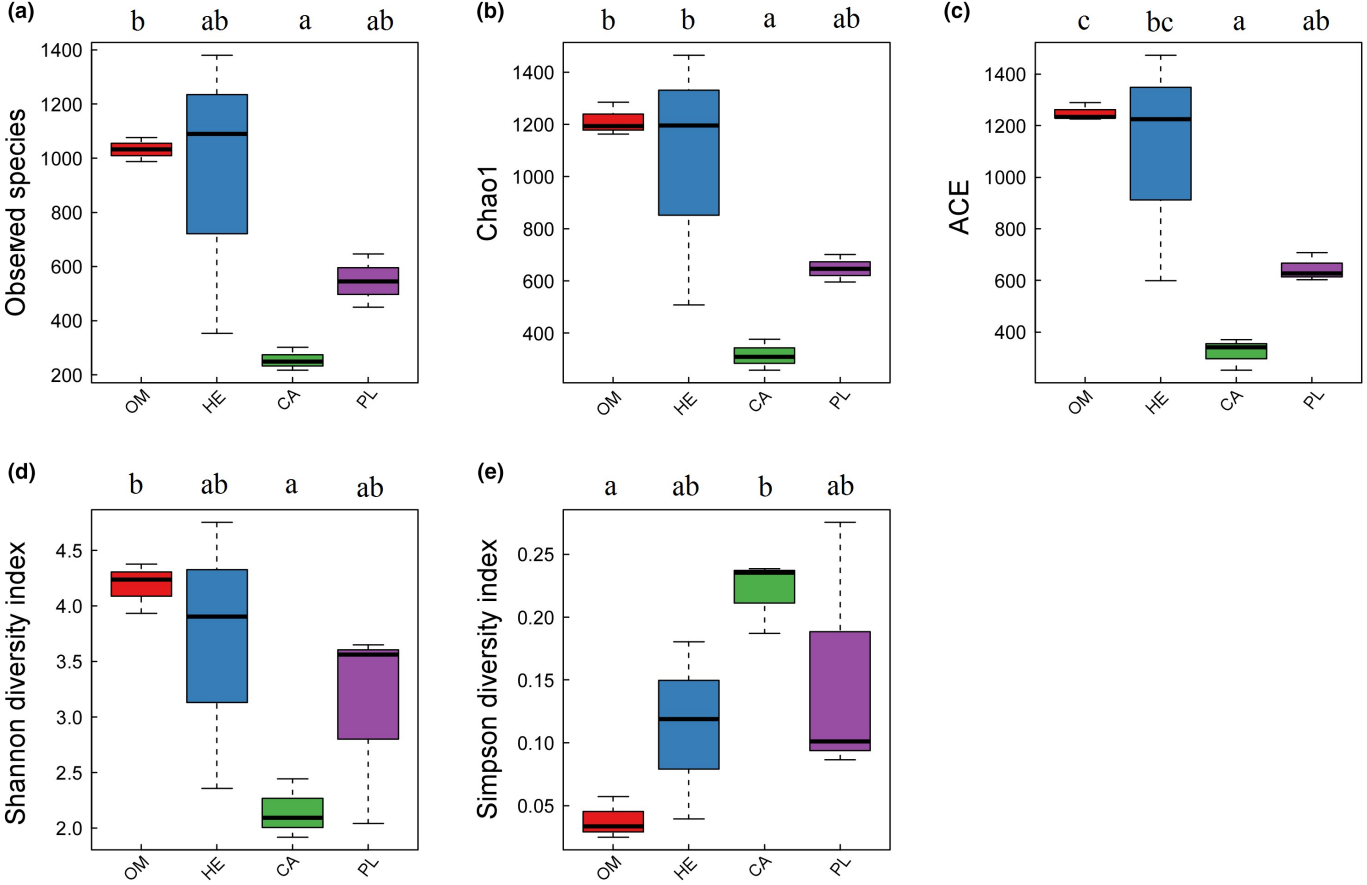
Intestinal microbiota abundance and diversity indices of the four fishes studied with different feeding habits. Bars with different letters indicate statistical difference (*p* < .05).

The results showed that the carnivorous fish species had significantly lower number of microbiota species than the omnivorous species (Figure 3a; *p* < .05). However, the carnivorous, herbivorous, and plankton feeder fish species had no significant difference in the number of microbiota species (*p* > .05). Similarly, the carnivorous and plankton feeder fish species had no significant difference in the number of microbiota species (*p* > .05). The carnivorous species also had significantly lower microbiota abundance as reflected by Chao1 (Figure 3b) and ACE (Figure 3c) than the omnivorous and herbivorous fish species (*p* < .05). The plankton feeder fish species also had significantly lower Chao1 than the omnivorous species (*p* < .05). However, the omnivorous, herbivorous, and plankton feeder species had statistically no difference in Chao1, while the herbivorous and plankton feeder species had no significant difference in ACE (*p* > .05). Similarly, the carnivorous and plankton feeder fish species had no significant differences in Chao1 and ACE (*p* > .05). The carnivorous fish species had significantly lower Shannon diversity index (Figure 3d), but higher Simpson's diversity index (Figure 3e) than the omnivorous fish species. However, the omnivorous, herbivorous, and plankton feeder fish species had no significant differences in Shannon diversity index and Simpson's diversity index (*p* > .05). Similarly, the carnivorous, herbivorous, and plankton feeder fish species had no significant differences in Shannon diversity index and Simpson's diversity index (*p* > .05). The community diversity of the four fish species studied followed the order omnivorous > herbivorous > plankton feeder > carnivorous.

### 3.5 Abundance and composition of microbiota at phyla and genera levels

**FIGURE 4:**
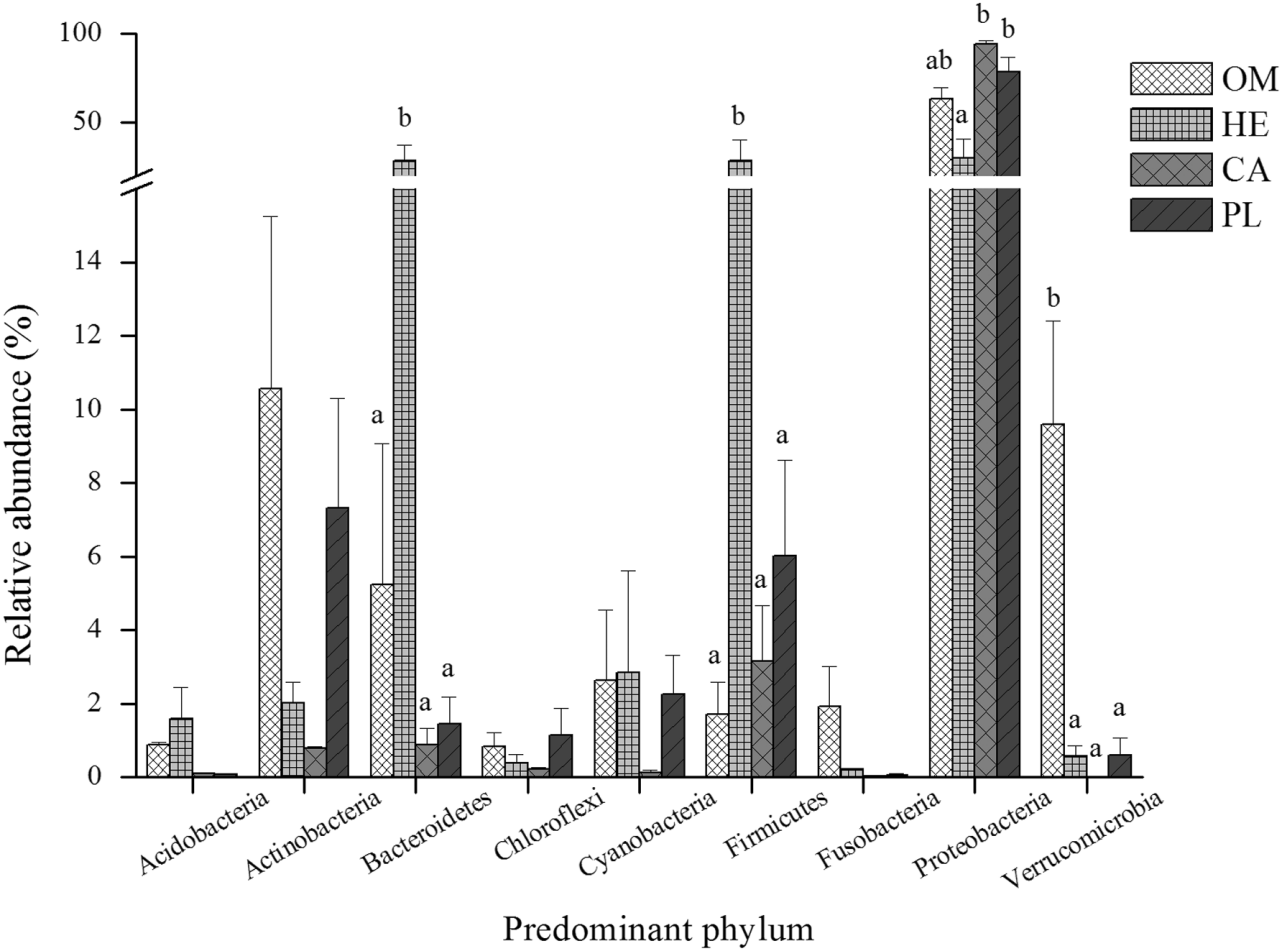
Abundance of fish intestinal dominant flora at phylum level for the four fishes studied. Different letters at each phylum indicate statistical difference (*p* < .05).

A total of 37 phyla were obtained from all the four fish species studied (Figure S1). We then selected the most abundant phyla with above 5% abundance. We obtained nine phyla classified as Proteobacteria, Firmicutes, Bacteroidetes, and Actinobacteria with relatively high abundance, representing 66.60%, 9.82%, 9.04%, and 5.18%, respectively (Figure 4). The herbivorous fish species had significantly lower Proteobacteria phylum abundance than the carnivorous and plankton feeder fish species (*p* < .05). On the contrary, the herbivorous fish species had significantly higher abundance of Firmicutes and Bacteroides phyla than the omnivorous, plankton feeder, and carnivorous fish species (*p* < .05). The omnivorous fish species had significantly higher *Verrucomicrobia* phylum abundance than the omnivorous, plankton feeder, and herbivorous fish species (*p* < .05).

A total of 324 bacterial genera were obtained. We then removed unidentified bacterial genera, and others and selected only those with more than 0.5% abundance. The results on composition showed 45 bacterial genera were obtained (Figure S2). The four fish species studied with different feeding habits had distinct microbiota composition at genera level for the nine phyla. The carnivorous and plankton feeder fish species were dominated by *Limnobacter* species (25.05% and 23.56%) and *Pseudomonas* species (26.07% and 8.16%), respectively. The microbiota in the omnivorous fish species was mainly composed of *Rhodobacter* species (14.99%), *Zymomonas* species (10.63%), *Clavibacter* species (8.57%), and *Luteolibacter* species (6.68%). The herbivorous fish species was mainly composed of *Bacteroides* species (22.56%) and *Citrobacter* species (9.43%).

We obtained 14 genera with significant differences in microbiota abundance (Table 3). The carnivorous fish species had significantly higher *Acinetobacter* species than the other three fish species studied (*p* < .05). The omnivorous fish species had higher abundance of *Anaerospora*, *Arenimonas*, *Dechloromonas*, *Deefgea*, *Luteolibacter*, and *Zymomonas* genera than the herbivorous and carnivorous fish species (*p* < .05). The herbivorous fish species had significantly higher *Bacteroides* species than the omnivorous, carnivorous, and plankton feeder fish species (*p* < .05). *Escherichia*, *Limnobacter*, and *Mycoplana* genera were abundant in the microbiota of the plankton feeder and carnivorous fish species, while *Pseudomonas* genus was abundant in the microbiota of plankton feeder and omnivorous fish species.

**TABLE 3:** Relative abundance of predominant genus in intestinal contents of herbivorous, plankton feeder, carnivorous, and omnivorous fish species studied.

Genus	Herbivorous	Plankton feeder	Carnivorous	Omnivorous	*p*‐value
*Acinetobacter*	0.616	0.355	2.612	0.162	.000
*Anaerospora*	0.054	0.174	0.008	1.602	.000
*Arenimonas*	0.040	0.008	0	1.097	.001
*Bacteroides*	22.558	0.937	0.170	0.079	.036
*Dechloromonas*	0.071	0.144	0.007	1.827	.001
*Deefgea*	0.015	0.012	0	0.431	.014
*Escherichia*	0.483	1.509	1.238	0.198	.000
*Limnobacter*	1.090	23.560	25.047	0	.000
*Luteolibacter*	0.129	0.422	0	6.676	.001
*Mycoplana*	0.063	1.239	2.199	0.013	.000
*Pseudomonas*	0.719	2.387	0.040	14.987	.000
*Rhodobacter*	3.784	0.793	0.025	0.093	.005
*Stenotrophomonas*	0.036	0.244	0.346	0.007	.010
*Zymomonas*	0.247	0.074	0.004	10.630	.007

### 3.6 LEfSe analysis of significantly enriched microbial communities

**FIGURE 5:**
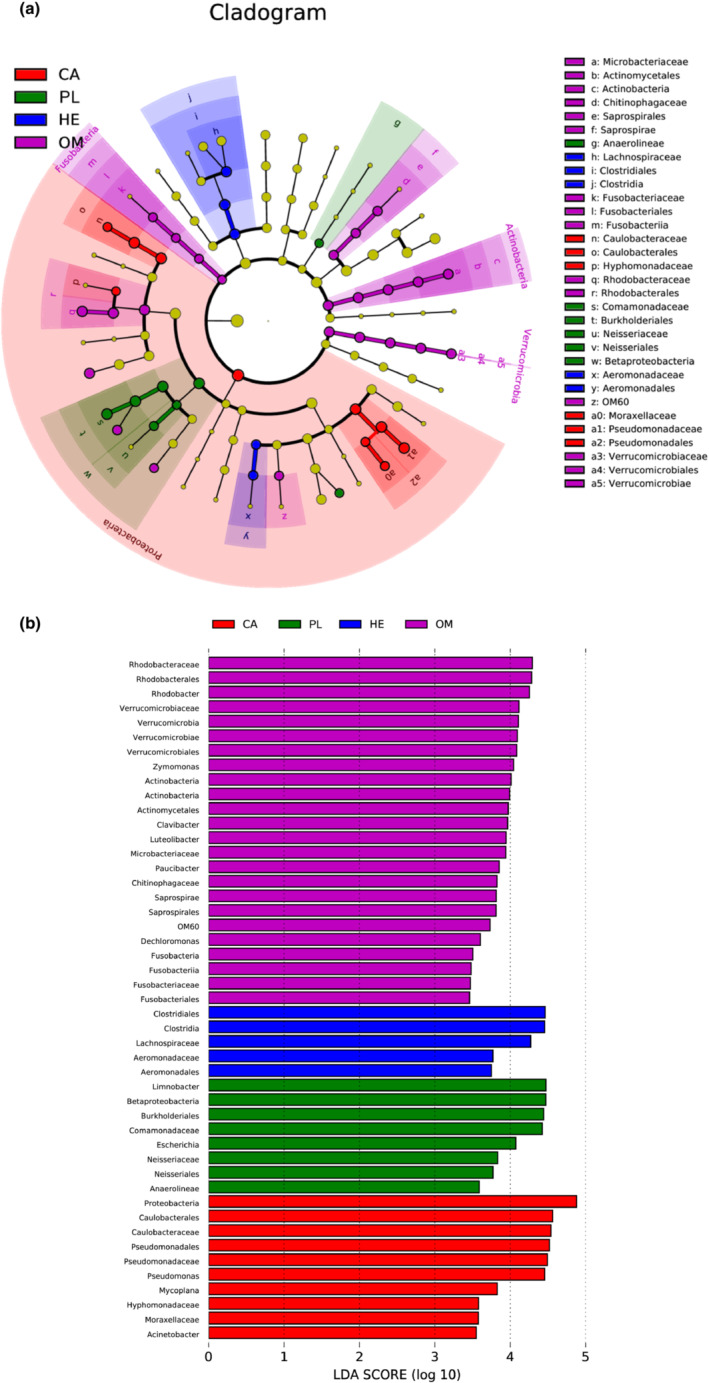
LEfSe analyses of gut microbial populations of the four fish species studied. Taxonomic cladogram of different microbial communities. (a) Identified differentially abundant taxa among four groups (HE, PL, OM, and CA) by linear discriminant analysis effect size (LEfSe) (log 10 ≥ 3.0). (b) Cladogram indicating LEfSe results presenting the recognized OTUs distributed according to phylogenetic characteristics around the circle. The dots in the center show the OTUs at phylum level, whereas the outer circle of dots indicates the OTUs at species level. The color of the dots and sectors present the most abundant OTUs in the four fish species with different feeding habits. Yellow color indicates OTUs with similar abundance in all compartments. The colored sectors give information on phylum (full name in the outermost circle, given only for phylum showing groups, class, order, and family that were significantly different among feeding habits are shown at the right side).

The LEfSe was used to characterize enriched microbial communities (Figure 5a). There were 48 differences among the four fish species studied with different feeding habits, classified from phylum to genus. The Proteobacteria phylum was common to all the four fish species studied. The omnivorous fish species enriched significantly higher gut microbiota species than the other three species studied. Indeed, the omnivorous fish species enriched significantly Fusobacteria, Actinobacteria, and Verrucomicrobia phyla compared with the carnivorous, herbivorous, and plankton feeder fish species. The most enriched bacteria in the gut of the four fish species studied followed the trend omnivorous (25) > carnivorous (10) > plankton feeder (8) > herbivorous (5) (Figure 5b). The omnivorous fish species enriched *Fusobacteriales*, *Fusobacteriaceae*, *Fusobacteriia*, *Fusobacteria*, *Dechloromonas*, *Saprospirales*, *Saprospirae*, *Chitinophagaceae*, *Paucibacter*, *Microbacteriaceae*, *Luteolibacter*, *Clavibacter*, *Actinomycetales*, *Actinobacteria*, *Actinobacteria*, *Zymomonas*, *Verrucomicrobiales*, *Verrucomicrobiae*, *Verrucomicrobia*, *Verrucomicrobiaceae*, *Rhodobacter*, *Rhodobacterales*, and *Rhodobacteraceae* genera. The herbivorous fish species enriched *Aeromonadales*, *Aeromonadaceae*, *Lachnospiraceae*, *Clostridia*, and *Clostridiales* genera. Moreover, the carnivorous fish species enriched *Acinetobacter*, *Moraxellaceae*, *Hyphomonadaceae*, *Mycoplana*, *Pseudomonas*, *Pseudomonadaceae*, *Pseudomonadales*, *Caulobacteraceae*, *Caulobacterales*, and *Proteobacteria* genera. The plankton feeder fish enriched *Anaerolineae*, *Neisseriales*, *Neisseriaceae*, *Escherichia*, *Comamonadaceae*, *Burkholderiales*, *Betaproteobacteria*, and *Limnobacter* genera.

### 3.7 Predicted gut microflora functions

**FIGURE 6:**
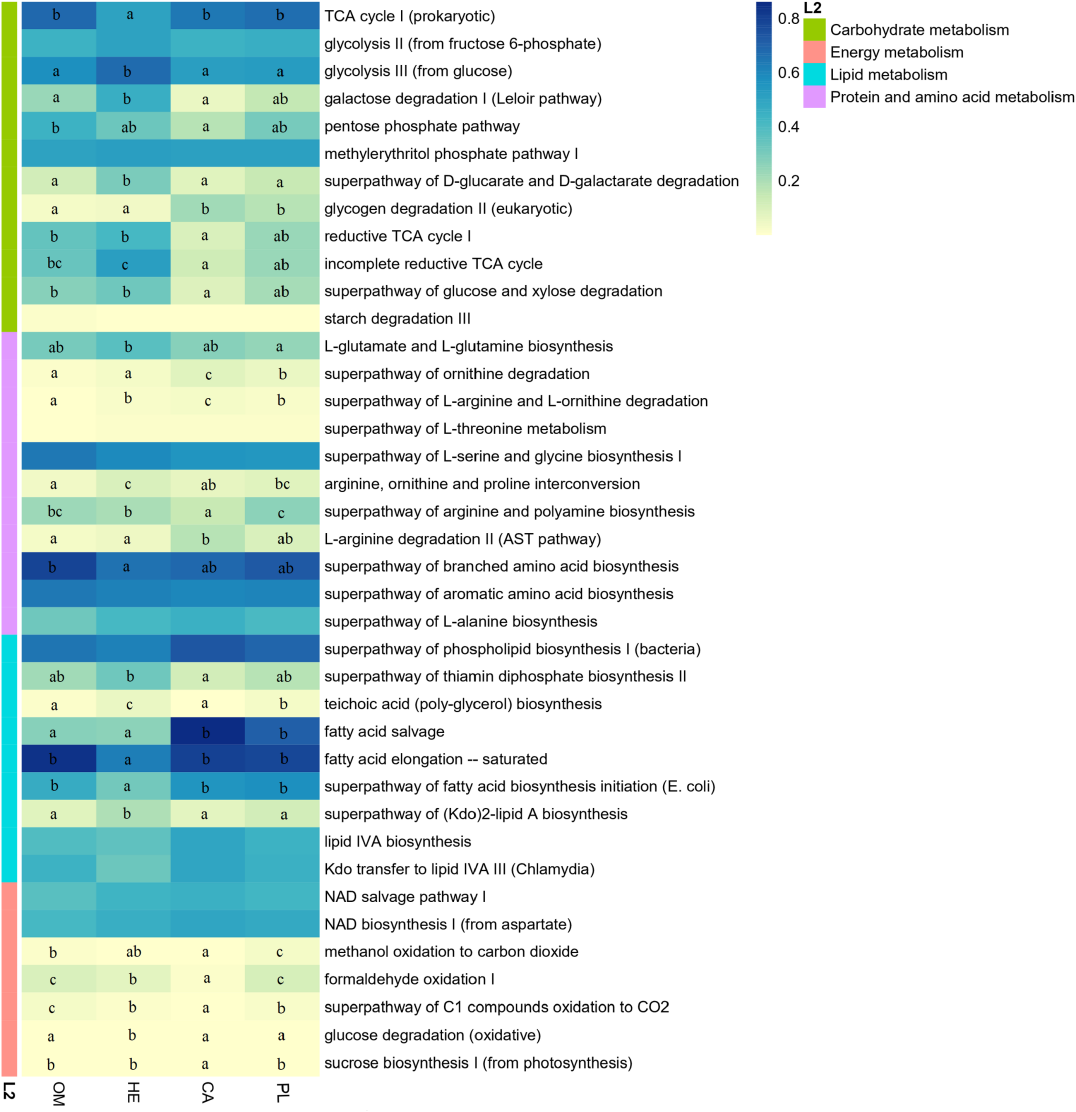
Heatmap presenting the abundance of digestion‐related bacterial gene functions among the four fishes studied with different feeding habits. Samples marked with different letters indicate significant differences (*p* < .05) among the four fish species studied with different feeding habits.

A total of 423 metabolism pathways were constructed. The four fish species studied showed marked differences in the functional profile (Figure S3). The microbial functions among the four fishes with different feeding habits showed that 39 pathways related to digestion were identified, including those associated with carbohydrate, protein and amino acids, energy, and lipid metabolism (Figure 6). Of all the pathways identified, 27 pathways were significantly changed (Figure 6). The herbivorous fish species had higher carbohydrate metabolism pathways than carnivorous fish species (*p* < .05). Moreover, the herbivorous fish species increased the pathways related to carbohydrate metabolism (i.e., glycolysis III [from glucose], galactose degradation I [Leloir pathway], superpathway of d‐glucarate and d‐galactarate degradation, reductive TCA cycle I and incomplete reductive TCA cycle) than the carnivorous fish species. Interestingly, the carnivorous fish species studied had more enriched protein and amino acid metabolism pathways (superpathway of ornithine degradation, superpathway of l‐arginine and l‐ornithine degradation and l‐arginine degradation II [AST pathway]) and lipid metabolism (fatty acid salvage) than the herbivorous and omnivorous fish species.

## 4 DISCUSSION

Fish feeding habit reflects directly the digestive ability to different nutrient components. The fish ability to digest and utilize different nutrients in feed is affected by the structure of the digestive tract, the digestive enzymes secreted and the intestinal microbiota composition, abundance, and diversity. This study explored the relationship between feeding habits of four fish species, which were omnivorous, carnivorous, herbivorous, and plankton feeder and digestive physiology, intestinal morphology, and intestinal microbial composition, abundance, and diversity. We also predicted the microbiome functions from the four fishes studied. We found clear differences in the digestive enzyme activities in the four fish species studied depending on their feeding habits. Evidently, the carnivorous fish species (*S. chuatsi*) had higher trypsin and pancrelipase activities in the hepatopancreas and enteropeptidase in the intestine than the herbivorous (*C. idella*), omnivorous (*C. auratus*) and the plankton feeder fish (*S. grahami*) species. The variations in digestive enzyme activities are caused by the different feeding habits (Xu et al., 2011). Accordingly, trypsin, intestinal enteropeptidase, and pancreatic lipase activities were roughly in the order carnivorous > omnivorous > herbivorous, reflecting the feeding on animal materials with high protein and lipid requiring secretions of related enzymes to digest them. Similarly, Liu et al. (2014) reported higher protease activity in carnivorous fish than omnivorous and herbivorous fish.

However, the intestinal lipase activity in this study was higher in the herbivorous and omnivorous fish species than the plankton feeder and carnivorous fish species studied. On the contrary, Parrizas et al. (1994) reported higher lipase activity in the carnivorous fish than the herbivorous and omnivorous fish. The higher lipase activity in the stomachless and herbivorous fish (*C. idellus*) is probably due to the relationship between intestinal tissue structure and digestive enzymes (Pan et al., 1996). In our study, the mandarin fish (*S. chuatsi*) represents a carnivorous fish species with a stomach, the grass carp (*C. idellus*) and Dianchi high‐back crucian carp (*C. auratus*) are typical herbivorous and omnivorous species, respectively, without stomach and, the Kunming Schizothoracin (*S. grahami*) is a plankton feeder fish with an enlarged sac between esophagus and intestinal tract, which can secrete digestive fluid and perform some stomach functions. Therefore, the higher intestinal lipase activity in the stomachless fish was due to enzyme secretions from the intestine, which performs some stomach functions of secreting enzymes. Interestingly, this study found higher pancreatic amylase and intestinal amylase activities in the herbivorous fish than the carnivorous fish species studied. Similarly, Li et al. (2012) found higher amylase in herbivorous than omnivorous fish and Liu and Zhang (2001) reported higher amylase activity in omnivorous fish than carnivorous fish. The higher amylase activity in the herbivorous fish studied is related to carbohydrate utilization of *C. idellus* in the diet. Similarly, omnivorous fish are capable of using higher carbohydrate levels than carnivorous fish (Li, Liu, et al., 2015).

This study found significant differences in intestinal VH, VW, and MT among the four fish species studied. These variations are due to the morphological and structural characteristics of the fish gut reflected by the different feeding habits (Liu & Zhang, 2001; Zeng & Ye, 1998). The intestinal microvilli of the studied carnivorous fish had many branches and complex structures. The microvilli complexity (VH and VW) and MT were in the order carnivorous > herbivorous > omnivorous of the studied fish species. The intestinal structure accommodates, transports, and digests feed and absorbs digested nutrients. The height, width, and intestinal microvilli complexity increase the surface area for ingested food digestion and absorption of the digested nutrients (Sun et al., 2019). The MT is composed of smooth muscle, which promotes food movement in the intestine through rhythmic relaxation and contraction. The muscle thickness layer directly reflects the contraction and peristalsis ability of the intestine. Accordingly, strengthening intestinal contraction and peristalsis is an effective means to increase feed digestion and reduce chyme circulation (Bian et al., 2021). Generally, it is known that carnivorous fish species have shorter intestines (Day et al., 2014). Accordingly, the increased intestinal structure complexity in the carnivorous fish studied reduces chyme circulation rate and enhances digested nutrient absorption. Therefore, our study indicates that the intestinal structure complexity is adapted to the fish feeding habit in order to fully achieve absorption of digested nutrients. However, our study analyzed intestinal morphology only in the midgut, which represents a limitation of the obtained results. Accordingly, future studies should analyze digestive enzymes in the foregut, midgut, and hindgut. Despite this limitation, the microvilli complexity and MT arranged in the order of carnivorous > herbivorous > omnivorous reflect the feeding habits of the four fish species studied for increased ingested food digestion and absorption of digested nutrients.

The vertebrate intestinal microflora play an important role in the host nutrition (Liu et al., 2016; Valdes et al., 2018). Previous studies have shown that dietary feeding habits (Miyake et al., 2015; Zhou et al., 2021) and host species (Li et al., 2019; Youngblut et al., 2019) are the main factors affecting the fish gut microbiota. This study also found that the fish gut microbiota diversity was affected significantly by the feeding habits and the host species. The lower microbiota abundance and diversity in the carnivorous fish studied reported in this study indicate nutritional instability because the fish gut microbiota abundance and diversity determine the host nutrition stability (Kuang et al., 2020) and higher Shannon index signifies better bacterial community stability and higher digestion of ingested nutrients (Zhang et al., 2019). Accordingly, the community diversity of the four fish species studied followed the order of omnivorous > herbivorous > plankton feeder > carnivorous. This order indicates that the carnivorous fish species studied had instable nutrient digestion, mainly digesting animal‐based materials. On the contrary, Li, Long, et al. (2015) reported higher bacterial diversity in the plankton feeder gut than the herbivorous fish.

This study showed Proteobacteria and Firmicutes phyla as typical dominant flora in the gut of the four fish species studied. Proteobacteria and Firmicutes are typical dominant flora in many fish intestine, such as *Oncorhynchus mykiss* (Ingerslev et al., 2014), *Nibea coibor* and *Nibea diacanthus* (Li et al., 2019), *Megalobrama terminalis* (Liu et al., 2021), *Micropterus salmoides* (Zhou et al., 2021), and *Symphysodon haraldi* (Zhang et al., 2021). The four fish species studied had variations in the microbiota abundance at genera level. However, our results on microbiota are limited by the lack of beta diversity analysis, an aspect requiring consideration in future studies. Nevertheless, the different symbiotic bacteria carried by the four fish species studied may be caused by the selective enrichment of different microorganisms due to variations in feeding habits and host species. A previous study indicated that during evolution, hosts tend to acquire suitable environmental bacteria by recognizing adhesion mechanisms on the cell surface (McFall‐Ngai, 2015). Clearly, the four fish species studied had relatively similar microbiota composition as other fish species.

Our study showed dominance of various genera in the different fish species studied, common to a notation that specific microbiota under different feeding conditions adapt to various functions (Rowland et al., 2018). For example, *Bacillus* species and *Cetacea* species are potential candidates for probiotics (Larsen et al., 2014), *Pseudomonas* species produces vitamin B_12_, and *Fusobacterium* species produces butyrate (Zhou et al., 2019). Accordingly, the *S. chuatsi* gut was dominated by *Acinetobacter* species, which may contribute to ingested protein digestion, while *Bacteroides* species were dominant in the herbivorous fish gut (*C. idella*), which may help the host to digest cellulose. The presence of this microbiome in the particular fish species is useful during host nutrition. Indeed, the intestinal microbiota in the carnivorous fish species (*S. chuatsi*) studied showed higher protein digestion and lower carbohydrate digestion, while the gut microbiota in the herbivorous fish (*C. idellus*) showed lower protein and higher carbohydrate digestion, consistent with their feeding habits.

## 5 CONCLUSION

Taken together, the digestive enzyme activities, intestinal morphology, and intestinal microbiome composition and diversity of the four fish species studied are affected significantly by their respective feeding habits. Accordingly, *S. chuatsi* as a carnivorous fish possesses higher trypsin and lipase activities related to its higher feeding habit on protein and lipid. On the contrary, the *C. idellus*, based on its herbivorous feeding habit of utilizing plant materials, has higher amylase enzyme activity. The intestinal microvilli of *S. chuatsi* has many branches and complex structures to increase surface area for digestion of ingested nutrients and absorption of digested nutrients as an adaption to the short intestine of a carnivorous fish and instable nutrition. The feeding habits led to various gut microbiota adaptations in the four fish species studied related to the selective colonization for various biological functions. Our results provide an understanding of the different digestive strategies of *C. auratus*, *S. chuatsi*, *C*. *idellus*, and *S. grahami* as representative species for omnivorous, carnivorous, herbivorous, and plankton feeder fish, respectively, for improving feed formulation. This is necessary for better feed utilization and digestibility in order to enhance digested nutrient absorption for promoting fish growth performance.

## AUTHOR CONTRIBUTIONS

**Fang Jiao:** Data curation (supporting); formal analysis (supporting); writing – original draft (lead); writing – review and editing (supporting). **Lei Zhang:** Data curation (lead); formal analysis (lead); software (lead). **Samwel Mchele Limbu:** Writing – review and editing (supporting). **Hong Yin:** Data curation (lead). **Yuqing Xie:** Data curation (supporting); software (lead). **Zihan Yang:** Data curation (supporting); methodology (supporting); software (supporting). **Zongmin Shang:** Methodology (supporting); project administration (supporting); software (supporting). **Lingfu Kong:** Formal analysis (supporting); resources (supporting); supervision (supporting). **Hua Rong:** Funding acquisition (lead); investigation (lead); writing – original draft (supporting); writing – review and editing (supporting).

## FUNDING INFORMATION

This study was financially supported by Guangdong Province Basic and Applied Basic Research Fund—Provincial and Municipal Joint Fund (2021A1515110179), Basic Research Program of Yunnan Province (202201AT070251), Yunnan Province Major Special Plan (202202AE090018), Natural Science Foundation of Hubei Province General Project (2023af) and Agricultural Basic Research Joint Special Project of Yunnan Province (2018FG001‐044).

## CONFLICT OF INTEREST STATEMENT

The authors declare that they have no conflicts of interest.

## Supporting information


Figure S1

Figure S2

Figure S3

## Data Availability

The data that support the findings of this study are available from the corresponding author upon a reasonable request.
