# Supplementary figures and images for: A comparison of digestive strategies for fishes with different feeding habits: Digestive enzyme activities, intestinal morphology, and gut microbiota
Source: Ecol Evol. 2023 Sep 12;13(9):e10499. doi: 10.1002/ece3.10499 (PMC10495811; doi:10.1002/ece3.10499)

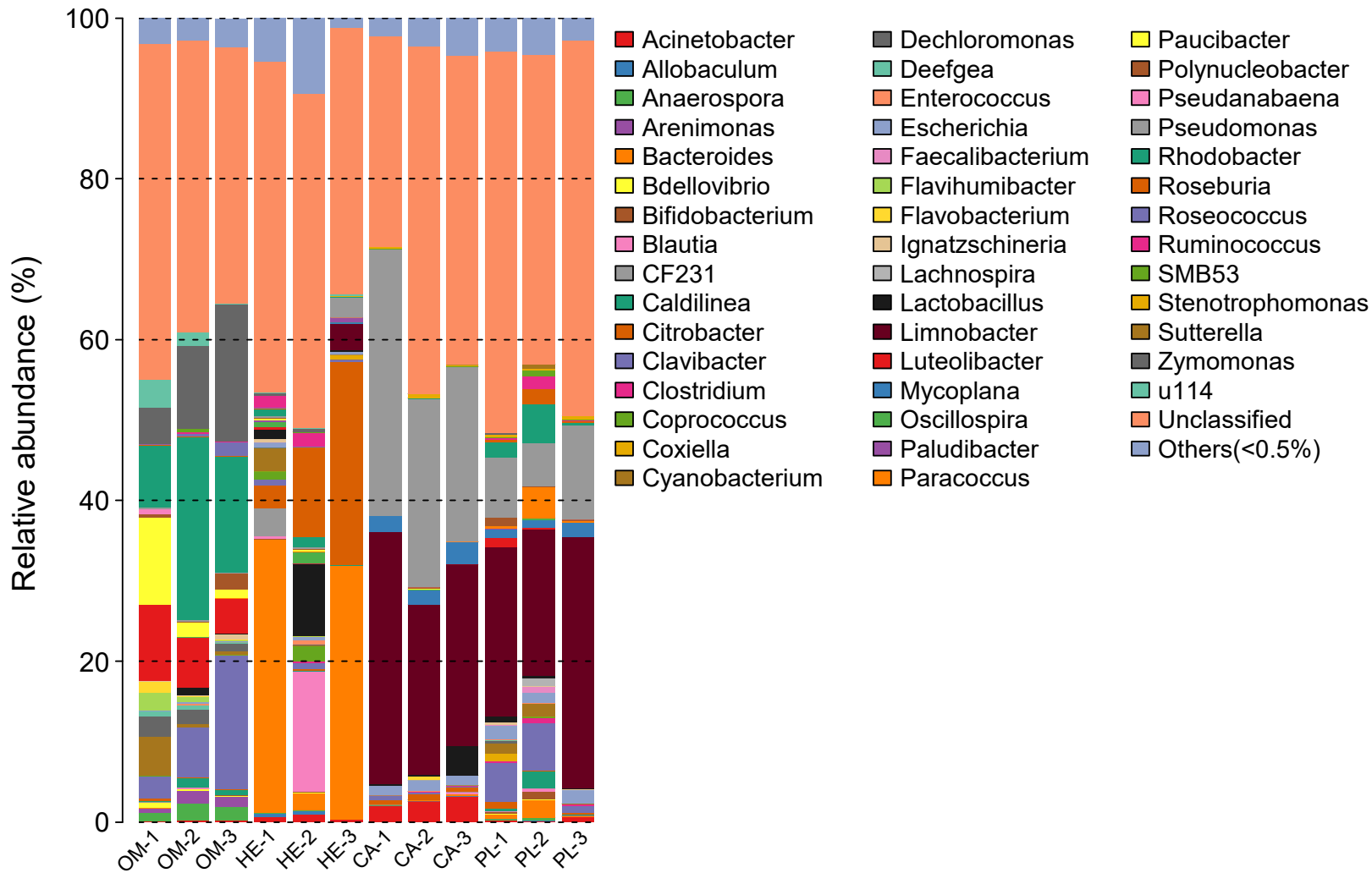

Supplement: Supplementary file 2 — Figure S2 [file ECE3-13-e10499-s003.pdf]

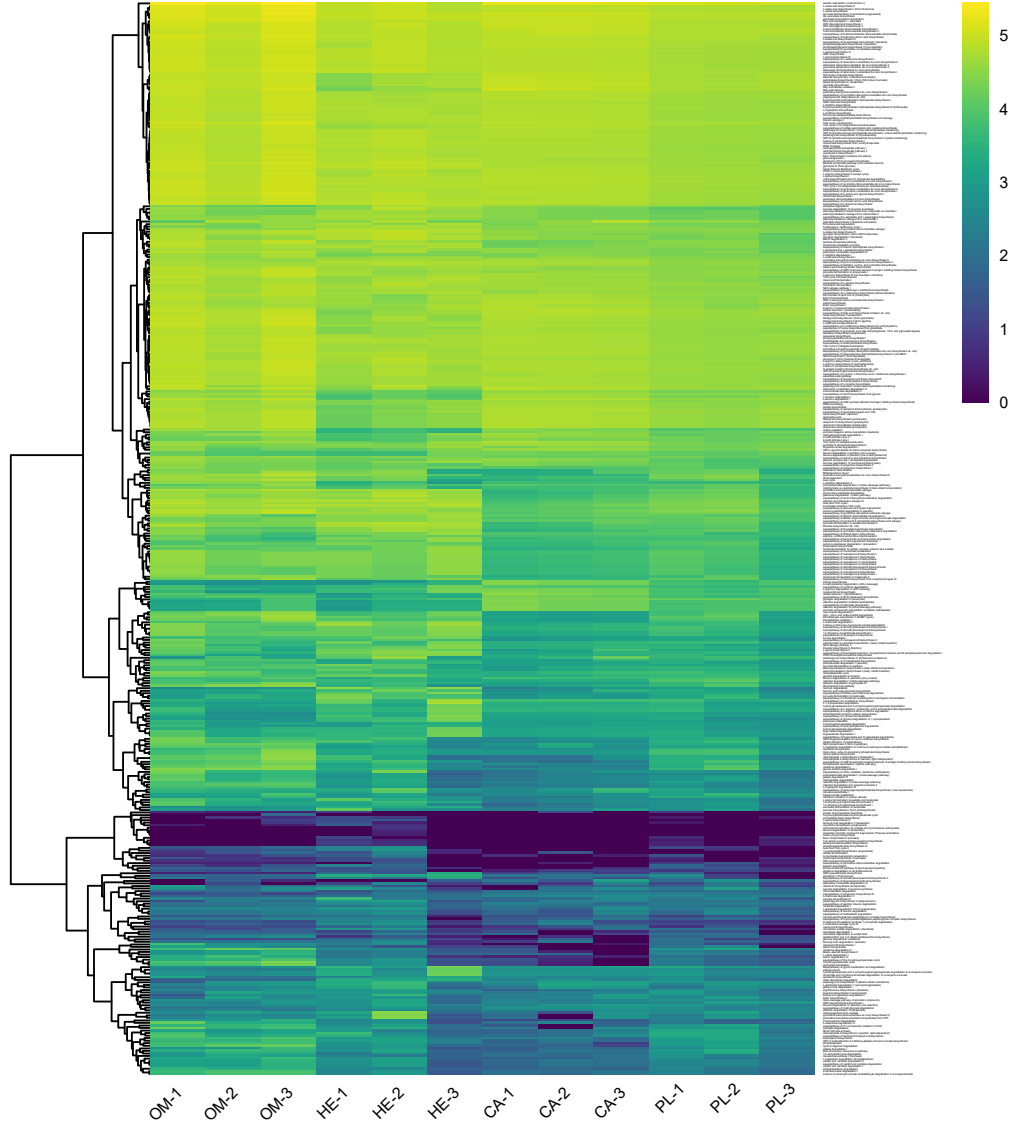

Supplement: Supplementary file 3 — Figure S3 [file ECE3-13-e10499-s002.pdf]
