# Supplementary material for: A comparison of digestive strategies for fishes with different feeding habits: Digestive enzyme activities, intestinal morphology, and gut microbiota
Source: Ecol Evol. 2023 Sep 12;13(9):e10499. doi: 10.1002/ece3.10499 (PMC10495811; doi:10.1002/ece3.10499)
Supplement: Supplementary file 1 — Figure S1 [file ECE3-13-e10499-s001.pdf]

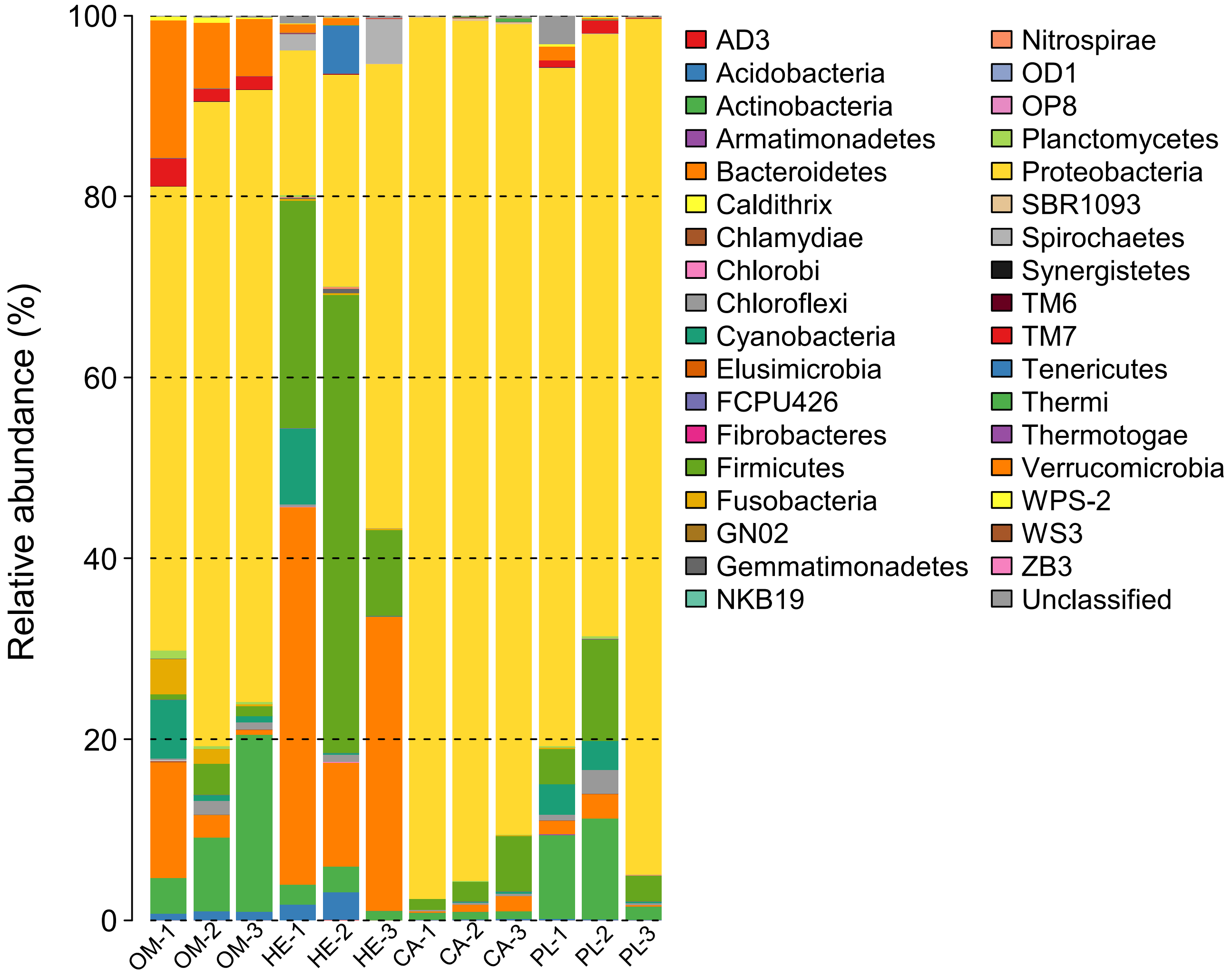

Relative abundance (%)
0
20
40
60
80
100
OM-1
OM-2
OM-3
HE-1
HE-2
HE-3
CA-1
CA-2
CA-3
PL-1
PL-2
PL-3
AD3
Acidobacteria
Actinobacteria
Armatimonadetes
Bacteroidetes
Caldithrix
Chlamydiae
Chlorobi
Chloroflexi
Cyanobacteria
Elusimicrobia
FCPU426
Fibrobacteres
Firmicutes
Fusobacteria
GN02
Gemmatimonadetes
NKB19
Nitrospirae
OD1
OP8
Planctomycetes
Proteobacteria
SBR1093
Spirochaetes
Synergistetes
TM6
TM7
Tenericutes
Thermi
Thermotogae
Verrucomicrobia
WPS-2
WS3
ZB3
Unclassified
